# Vaccination with transgenic *Eimeria tenella* expressing *Eimeria maxima* AMA1 and IMP1 confers partial protection against high-level *E. maxima* challenge in a broiler model of coccidiosis

**DOI:** 10.1186/s13071-020-04210-2

**Published:** 2020-07-10

**Authors:** Iván Pastor-Fernández, Sungwon Kim, Virginia Marugán-Hernández, Francesca Soutter, Fiona M. Tomley, Damer P. Blake

**Affiliations:** 1 Department of Pathobiology and Population Sciences, Royal Veterinary College, Hawkshead Lane, Hatfield, Hertforshire AL9 7TA UK; 2 SALUVET, Animal Health Department, Faculty of Veterinary Sciences, Complutense University of Madrid, 28040 Madrid, Spain

**Keywords:** Poultry coccidiosis, Vaccination, Transgenic *Eimeria tenella*, Apical Membrane Antigen-1, Immune Mapped Protein-1, Broiler model of coccidiosis, Productive scores

## Abstract

**Background:**

Poultry coccidiosis is a parasitic enteric disease with a highly negative impact on chicken production. In-feed chemoprophylaxis remains the primary method of control, but the increasing ineffectiveness of anticoccidial drugs, and potential future restrictions on their use has encouraged the use of commercial live vaccines. Availability of such formulations is constrained by their production, which relies on the use of live chickens. Several experimental approaches have been taken to explore ways to reduce the complexity and cost of current anticoccidial vaccines including the use of live vectors expressing relevant *Eimeria* proteins. We and others have shown that vaccination with transgenic *Eimeria tenella* parasites expressing *Eimeria maxima* Apical Membrane Antigen-1 or Immune Mapped Protein-1 (*Em*AMA1 and *Em*IMP1) partially reduces parasite replication after challenge with a low dose of *E. maxima* oocysts. In the present study, we have reassessed the efficacy of these experimental vaccines using commercial birds reared at high stocking densities and challenged with both low and high doses of *E. maxima* to evaluate how well they protect chickens against the negative impacts of disease on production parameters.

**Methods:**

Populations of *E. tenella* parasites expressing *Em*AMA1 and *Em*IMP1 were obtained by nucleofection and propagated in chickens. Cobb500 broilers were immunised with increasing doses of transgenic oocysts and challenged two weeks later with *E. maxima* to quantify the effect of vaccination on parasite replication, local IFN-γ and IL-10 responses (300 oocysts), as well as impacts on intestinal lesions and body weight gain (10,000 oocysts).

**Results:**

Vaccination of chickens with *E. tenella* expressing *Em*AMA1, or admixtures of *E. tenella* expressing *Em*AMA1 or *Em*IMP1, was safe and induced partial protection against challenge as measured by *E. maxima* replication and severity of pathology. Higher levels of protection were observed when both antigens were delivered and was associated with a partial modification of local immune responses against *E. maxima*, which we hypothesise resulted in more rapid immune recognition of the challenge parasites.

**Conclusions:**

This study offers prospects for future development of multivalent anticoccidial vaccines for commercial chickens. Efforts should now be focused on the discovery of additional antigens for incorporation into such vaccines.
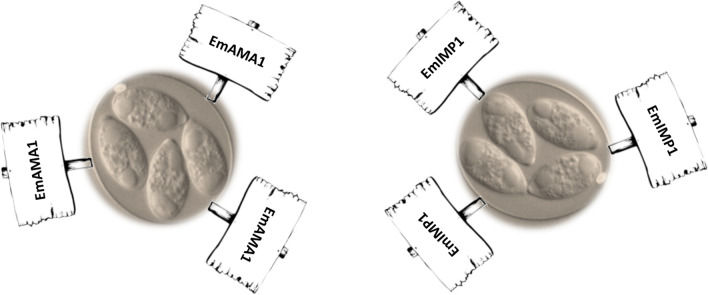

## Background

The genus *Eimeria* includes a large number of species, many of which can cause the disease coccidiosis in domestic livestock. Infection results in clinical or subclinical enteritis, typically self-limiting, but often with a negative impact on key production parameters [1]. Current intensive husbandry practices in poultry production systems provide an ideal environment for *Eimeria* transmission, transforming coccidiosis into a major problem that has been associated with annual global costs in excess of £2 billion [1–3]. Management of variables such as poultry stocking density, quality of housing and ventilation can reduce *Eimeria* transmission, but additional anticoccidial control is still essential [4]. In-feed chemoprophylaxis remains the primary method of control [5], although resistance has been described among *Eimeria* to every drug currently available [6]. Vaccination using formulations of live *Eimeria* parasites offers an effective alternative to chemoprophylaxis, although the occurrence of multiple *Eimeria* species that infect chickens and the lack of cross-protective immunity between them requires vaccines to include lines of most, if not all *Eimeria* species [4]. The expansion of ‘no antibiotics, ever’ production systems has encouraged increased use of non-attenuated, wild-type vaccines in countries such as the USA, but uptake of safer, live-attenuated vaccines remains limited to the minority layer and breeder sectors in most countries. Availability of commercial live-attenuated vaccines is constrained by limitations in the capacity of their production, as each vaccine line requires independent passage through chickens, incurring costs that are significantly higher than for routine chemoprophylaxis or for non-attenuated vaccines. In the broiler sector, where profit margins are very tight, control measures are still highly dependent on the use of anticoccidial drugs, but these are increasingly ineffective or may become restricted in the near future [4, 7]. Therefore, there is an urgent need to reduce the cost and improve the availability of anticoccidial vaccine formulations to make them more attractive for this sector.

To date, several *Eimeria* proteins with relevant roles in host/parasite interaction have been tested as anticoccidial vaccines in diverse formulations, with varying efficacies [4, 8, 9]. Many of these antigens have not been developed further as vaccines, in part because they have not met what has been regarded as sufficient immune protection against challenge and/or because of the need for multiple rounds of vaccination. However, several studies have achieved levels of immune protection approaching those reported for the ionophores and for live vaccines when they were first developed (e.g. an ~ 60–90% reduction in parasite replication). Both of these well-established methods for controlling coccidiosis work so well because they allow low levels of *Eimeria* replication to continue, thus providing natural boosting of protective immunity as the parasites that escape the effects of treatment recycle through the chickens [8, 10]. On this basis, we have hypothesised that the use of live replicating vector systems expressing previously tested *Eimeria* antigens, could work well for automated single-shot anticoccidial vaccine delivery, despite conferring less than complete protection against challenge. Following this hypothesis, we and others have recently shown that *Eimeria tenella* parasites can be used as a vector to express and deliver the protein Apical Membrane Antigen 1 from *Eimeria maxima* (*Em*AMA1), and that vaccination with such parasites was sufficient to induce significant partial protection against challenge with *E. maxima* oocysts [11]. Similar results were reported with *E. tenella* parasites expressing Immune Mapped Protein-1 from *E. maxima* (*Em*IMP1) [12], and more recently with a combination of *Em*AMA1 and *Em*IMP1-expressing parasites [13]. However, these studies were performed in inbred chicken lines kept in wire-floor cages and challenged with low parasite doses, so the data cannot be directly related to a farm setting where outbred chickens are repeatedly exposed to recycling vaccine parasites as well as to higher challenge doses of virulent wild type oocysts. Alone, low-dose challenges are not suitable for evaluation of factors relevant to a commercial perspective such as protection against intestinal damage and body weight gain.

The present study aimed to evaluate the suitability of *E. tenella* parasites expressing *Em*AMA1 or *Em*IMP1 proteins to induce significant levels of cross-protection against *E. maxima* under commercial conditions. For this purpose, Cobb500 broiler chickens were vaccinated with increasing doses of transgenic parasites to mimic natural recycling, reared in floor pens at commercial-level stocking densities, and subsequently challenged with a dose of pathogenic *E. maxima* oocysts (10,000) to assess vaccine efficacy in terms of lesion scores (protection against parasite-induced pathology) and body weight gain (protection against compromised growth). In an effort to correlate these parameters with levels of parasite replication, a subgroup of vaccinated broilers were challenged with a low dose of *E. maxima* oocysts (300) and used to quantify the effect of vaccination on local parasite burdens by quantitative PCR. Here we demonstrate that vaccination with transgenic *E. tenella* oocysts expressing *Em*AMA1 or with a mix of oocysts expressing either *Em*AMA1 or *Em*IMP1 induces a significant reduction in parasite replication, alleviates lesion scores and ameliorates reduction in body weight gain due to *E. maxima* challenge.

## Methods

### Parasite passage

Four weeks old Lohmann Selected Leghorn (LSL) chickens reared under specific pathogen-free conditions were used to propagate oocysts of the Wisconsin (Wis) strain of *E. tenella* and the Weybridge (W) strain of *E. maxima* as previously described [14]. Standard methods were used to recover and sporulate oocysts and to purify sporozoites through nylon wool and DE-52 columns [15, 16].

### Preparation of transgenic *E. tenella* Wis parasites expressing *Em*AMA1 and *Em*IMP1

*Eimeria tenella* Wis parasites expressing *Em*AMA1 (termed *Et*[*Em*AMA1]) and parasites expressing only delivery signals (*Et*[GPI], empty vector) were used as previously described [11, 17]. Similar procedures were carried out to obtain *E. tenella* parasites expressing *Em*IMP1. Briefly, the *Em*IMP1 coding sequence (GenBank: KP642747.1) was amplified from the pET32b-*Em*IMP1 plasmid [18] and flanked with *Xba*I restriction sites by PCR using Platinum *Taq* DNA Polymerase High Fidelity® (Invitrogen, Paisley, UK) with the following primers (restriction sites underlined): 5′-GCT CTA GAG GGG CCG CTT GCG GGA AA-3′ and 5′-GCT CTA GAA TCT TGC GAC ACT TTA GT-3′ (Sigma-Aldrich, Gillingham, UK). The *Em*IMP1 sequence was subsequently cloned into the *Xba*I site of the core construct used for *E. tenella* transfection, which contains (i) the mCitrine reporter and (ii) the mCherry reporter, preceded by the *Xba*I restriction site and flanked with the signal peptide of the EtMIC2 protein (SP2) and the glycosylphosphatidylinositol anchor of the EtSAG1 protein (GPI) [17]. Additionally, a plasmid carrying the mutant *Toxoplasma gondii* dihydrofolate reductase-thymidylate synthase (DHFR-TSm2m3) gene that confers resistance to pyrimethamine was also prepared for co-transfection [19]. Final plasmids were prepared for transfection using a Midi Prep Kit (Qiagen, Manchester, UK), digested for linearisation with *Psi*I (New England BioLabs, Hitchin, UK), precipitated in ethanol-sodium acetate and quantified by NanoDrop (Thermo Fisher Scientific, East Grinstead, UK). A total of 1 × 10^6^ freshly hatched *E. tenella* Wis sporozoites were transfected in duplicate with 12 µg (*Em*IMP1) and 4 µg (DHFR-TSm2m3) of *Psi*I-digested plasmids together with 6 U of *Psi*I in Lonza buffer P3 using the programme EO114 of the Nucleofector 4D (Lonza, Basel, Switzerland). After shock, parasites were left for 20 min at room temperature in Roswell Park Memorial Institute (RPMI) medium (Sigma-Aldrich), pooled and used to infect two four week-old LSL chickens by the cloaca (0.75 × 10^6^ sporozoites/bird). One day after infection, birds were in-feed supplemented with pyrimethamine for 6 days (150 ppm, Sigma-Aldrich) [19]. Seven days after infection, oocysts were harvested, sporulated and used for subsequent *in vivo* passage after population enrichment for fluorescent parasites by fluorescence-activated cell sorting (FACS) (FACS Aria III, BD) [20].

Transcription of the *EmIMP1* gene was confirmed using transgenic populations by reverse transcription (RT) PCR with the primers: 5′-CAT TCA CCT TAC ACC ACT TTG-3′ (Fw_EmIMP1-int, which anneals to the residues 692–712 of the *Em*IMP1-coding sequence) and 5′-ATG GTC TTC TTC TGC ATT ACG-3′ (Rv_mCherry-int, which anneals to the residues 423–443 of the mCherry-coding sequence). For this purpose, total RNA was extracted from populations of transfected oocysts using the TRIzol® reagent (Invitrogen) and complementary DNA (cDNA) was generated using SuperScript II® reverse transcriptase and random hexamer primers (Invitrogen) as previously described [20]. The absence of genomic DNA contamination was confirmed by PCR targeting the *E. tenella* actin locus with primers that amplify a region coded between two adjacent exons as described earlier [11]. Expression of the *Em*IMP1 protein in transgenic parasites was confirmed by fluorescent microscopy through detection of the mCherry tag with a SP5 confocal microscope (Leica Microsystems, Wetzlar, Germany). Image processing was performed using ImageJ software (NCBI, http://rsb.info.nih.gov/ij/).

### *In vivo* immunisation trial of *E. tenella* Wis parasites expressing *Em*AMA1 and *Em*IMP1

A total of 144 Cobb500 broiler chicks (PD Hook hatcheries) vaccinated against infectious bronchitis virus (IB H120 vaccine) were purchased at day of hatch (day 0), weighed and distributed evenly into six different groups of 24 in independent wire-floored cages (Table 1). In order to mimic parasite recycling and ensure solid immunity (the so-called trickle infection, [21]), chicks from groups 3 to 6 were immunised by oral gavage with 100, 500 and 3000 sporulated oocysts at days 2, 8 and 14 of age, respectively; chicks from groups 1 to 2 were inoculated with sterile water (Table 1). At days 10, 16 and 22 (8 days after each immunisation), faecal samples were randomly collected from the bottom of all cages to confirm cycling of vaccine lines by oocyst flotation [14]. Parallel analyses also confirmed that groups 1 and 2 remained non-infected during the same period. At 15 days of age, 18 out of 24 birds from each group were transferred to floor pens at high stocking densities (~ 650 cm^2^/bird), whereas 6 birds were kept in the original cages. At 29 days of age, 15 days after the last immunisation, birds from groups 2 to 6 were challenged with freshly harvested *E. maxima* W oocysts (1-month-old). Two different challenge doses were employed: birds kept in cages (*n* = 6/group) were challenged with 300 oocysts in order to quantify the effect of vaccination on parasite replication; birds kept in floor pens (*n* = 18/group) were infected with 10,000 oocysts to assess if vaccination was able to protect against compromised body weight gain and development of intestinal lesions; all birds from group 1 were dosed with sterile water. At day 35, 6 days after challenge, all birds kept in cages and infected with 300 oocyst/bird were culled by cervical dislocation and the middle section of the intestine (~ 5 cm around Meckel’s diverticulum, representing the terminal jejunum and proximal ileum) was collected and preserved in RNAlater at − 20 °C (Thermo Fisher Scientific) until further analysis. On the same day, 7 out of 18 birds kept in floor pens and infected with 10,000 oocysts/bird were also culled to determine intestinal lesion scores following standard procedures [22]. In order to quantify body weight gains, the remaining birds (11 birds/group; 10,000 oocysts/bird) were kept in floor pens until 41 days of age, 12 days after challenge.

**Table 1:** Experimental design for vaccine trial

Group	Abbreviation	Vaccine	Immunisation protocol			Challenge (*E. maxima* W)	*n* PR^d^ (cages)	*n* LS^e^ (pens)	*n* BWG^f^ (pens)
			Day 2	Day 8	Day 14	Day 29	Culled at day 35	Culled at day 35	Culled at day 41
1	H_2_O-H_2_O	H_2_O	H_2_O	H_2_O	H_2_O	H_2_O	–	7	11
						H_2_O	6	–	–
2	H_2_O-Emax	H_2_O (no protection)	H_2_O	H_2_O	H_2_O	10,000	–	7	11
						300	6	–	–
3	Emax-Emax	*E. maxima* W (‘full’ protection)	100	500	3000	10,000	–	7	11
						300	6	–	–
4	*Et*[GPI]-Emax	*Et*[GPI]^a,b^ (empty vector)	100	500	3000	10,000	–	7	11
						300	6	–	–
5	*Et*[A]-Emax	*Et*[*Em*AMA1]^a^	100	500	3000	10,000	–	7	11
						300	6	–	–
6	*Et*[A + I]-Emax	*Et*[*Em*AMA1] + *Et*[*Em*IMP1]^a,c^	100	500	3000	10,000	–	7	11
						300	6	–	–

^a^FACS enriched transgenic *E. tenella* parasites

^b^*Eimeria tenella* Wis parasites expressing the signal peptide of the EtMIC2 protein and the glycosylphosphatidylinositol (GPI) anchor of the EtSAG1 protein

^c^Equal proportions of *Em*AMA1 and *Em*IMP1-expressing parasites were used for vaccination

^d^Number of birds used to quantify parasite replication

^e^Number of birds used to assess lesion scores

^f^Number of birds used to quantify body weight gains

Chickens from all groups were weighed throughout the experiment at 2 (before first vaccination), 29 (before challenge), 35 (6 days after challenge) and 41 days of age (12 days after challenge). Body weight gains (BWG) were calculated as follows: %BWG = (Final weight − Initial weight)/(Initial weight) × 100. Water and anticoccidial-free feed (baby chick crumbs, SmallHolder range) were provided *ad libitum* throughout the trial.

Data were analysed using GraphPad Prism version 7.02. Data normality was confirmed with the Shapiro-Wilk test. One-way ANOVA with a Tukey’s *post-hoc* test was used to compare BWG and parasite replication values. Kruskal-Wallis with a Dunn’s *post-hoc* test was performed to analyse differences in lesion scores.

### DNA and RNA extractions

Intestinal samples from all chickens challenged with 300 oocysts were removed from RNAlater solution, weighed and disrupted with the TissueRuptor homogenizer (Qiagen) in RLT plus lysis buffer (Qiagen) supplemented with 1% 2-mercaptoethanol (Sigma-Aldrich) at a ratio of 600 µl buffer per mg of tissue. A total of 30 mg of homogenate (~ 450 µl) were further homogenised using QIAshredder columns (Qiagen) and subsequently employed for simultaneous purification of DNA and RNA using the AllPrep DNA/RNA Mini Kit (Qiagen) following the manufacturer’s guidelines. DNA and RNA quality was checked by agarose gel electrophoresis and using a NanoDrop Spectrophotometer (Thermo Fisher Scientific).

### Quantification of *E. maxima* replication

Plasmids harbouring fragments of the *E. maxima* MIC1 (*Em*MIC1) and the chicken beta-actin (*Gg*ACTb) genes were used as single copy template positive controls [23, 24]. The pGEMT-*Em*MIC1 plasmid was obtained from a previous study [23], whereas the pGEMT-*Gd*ACTb was obtained as follows: a 958 bp fragment of the *Gd*ACTb genomic sequence was amplified by PCR from chicken genomic DNA using the Platinum Taq DNA Polymerase High Fidelity® (Invitrogen) and the primers 5′-CTA GAG GAG CAG AGA AGC CTC TTA-3′ and 5′-CTAGAGGAGCAGAGAAGCCTCTTA-3′ (derived from Accession Number X00182.1, purchased from Sigma-Aldrich). The PCR product was cloned using the pGEM®-T Easy Vector System (Promega, Hampshire, UK), propagated in *E. coli* XL1-Blue competent cells (Stratagene, California, USA), purified using the QIAprep Spin Miniprep Kit (Qiagen) and sequenced (GATC Biotech, Konstanz, Germany). Ten-fold dilution series representing 10^6^ to 10^0^ copies of each plasmid were prepared using glycogen as a carrier (final concentration of 33 μg/ml, Thermo Fisher Scientific) as described previously [23, 24].

Quantitative real-time PCR (q-PCR) was performed as previously described [24] using the primers listed in Table 2. All reactions were conducted employing white Hard-shell® 96-well PCR plates and the CFX96 Touch® Real-Time PCR Detection System (Bio-Rad Laboratories, Hertfordshire, UK). Intestinal DNA samples were amplified in triplicate in a 20 μl reaction containing 1 μl of total gDNA, 300 nM of each primer, 10 μl of SsoFast™ EvaGreen® Supermix (Bio-Rad Laboratories) and 8.5 μl of DNase/RNase free water (Thermo Fisher Scientific). Thermocycling conditions consisted of 95 °C for 2 min, followed by 40 cycles of 95 °C for 15 s and 60 °C for 30 s with a subsequent melt analysis of 65 °C–95 °C at increments of 0.5 °C/0.5 s. Each assay included the relevant plasmid standards and no template controls. The number of genomes from the host (*Gd*ACTb target) and the *E. maxima* parasites (*Em*MIC1 target) were estimated by comparison with the plasmid standard series. Triplicate data arising from each test sample were averaged and standardised by comparison with host genome concentration as *E. maxima* genomes/host genomes ratio. Data normality was confirmed with the Shapiro-Wilk test and subsequently analysed by one-way ANOVA with a Tukey’s *post-hoc* test using GraphPad Prism version 7.02.

**Table 2:** Primer sequences used for q-PCR analyses

Gene	Forward primer (5′-3′)	Reverse primer (5′-3′)	GenBank ID	PMID
*GgACTb*	GAGAAATTGTGCGTGACATCA	CCTGAACCTCTCATTGCCA	X00182.1	26141544
*EmMIC1*	TCGTTGCATTCGACAGATTC	TAGCGACTGCTCAAGGGTTT	M99058	16300767
*Gg28S* rRNA	GGCGAAGCCAGAGGAAACT	GACGACCGATTTGCACGTC	AH001604	25796577
*GgIFNγ*	GCTCCCGATGAACGACTTGA	TGTAAGATGCTGAAGAGTTCATTCG	GQ421600.1	20470818
*GgIL10*	CATGCTGCTGGGCCTGAA	CGTCTCCTTGATCTGCTTGATG	NM_001004414	29316981

### Quantification of local IFN-γ and IL-10 expression

Transcription of IFN-γ and IL-10 was analysed by RT-q-PCR as an indication of expression as previously described [25] using RNA extracted from intestinal samples (see above). Briefly, a total of 1 µg RNA was used to synthesise complementary DNA (cDNA) using the iScript™ cDNA Synthesis Kit (Bio-Rad Laboratories) as indicated by the manufacturers. Synthesized cDNA was diluted in DNase/RNase-Free Water as follows: 1:100 for *28S* rRNA quantification, 1:5 for *IFN-γ* quantification and no dilution for *IL-10* transcripts.

RT-q-PCR reaction mixture was prepared with 1 µl of cDNA, 500 nM of each primer (Table 2), 5 µl of 2× SsoFast™ EvaGreen® Supermix (Bio-Rad Laboratories) and 3 µl of DNase/RNase-Free Water in a final volume of 10 µl per reaction. Ten-fold dilution series for target genes (*28S* rRNA, *IFN-γ* and *IL-10*) were prepared from a pool of cDNA samples obtained from all analysed chickens. All samples and standard points were analysed in duplicate with pertinent non-template controls under the following thermocycling conditions: 95 °C for 2 min, followed by 40 cycles of 95 °C for 15 s and 60 °C for 30 s with a subsequent melt analysis of 65–95 °C at increments of 0.5 °C/0.5 s. Data were normalised using the *28S* rRNA target, represented as corrected 40-Ct values. For statistical analyses, data normality was confirmed with the Shapiro-Wilk test and compared by one-way ANOVA with a Tukey’s *post-hoc* test using GraphPad Prism version 7.02. Two-tailed Pearson’s correlation coefficients between IFN-γ and IL-10 expression levels and parasite replication scores were also calculated using the same software.

## Results

### Transcription and expression of *Em*IMP1 in transgenic *E. tenella* parasites

**Fig. 1:**
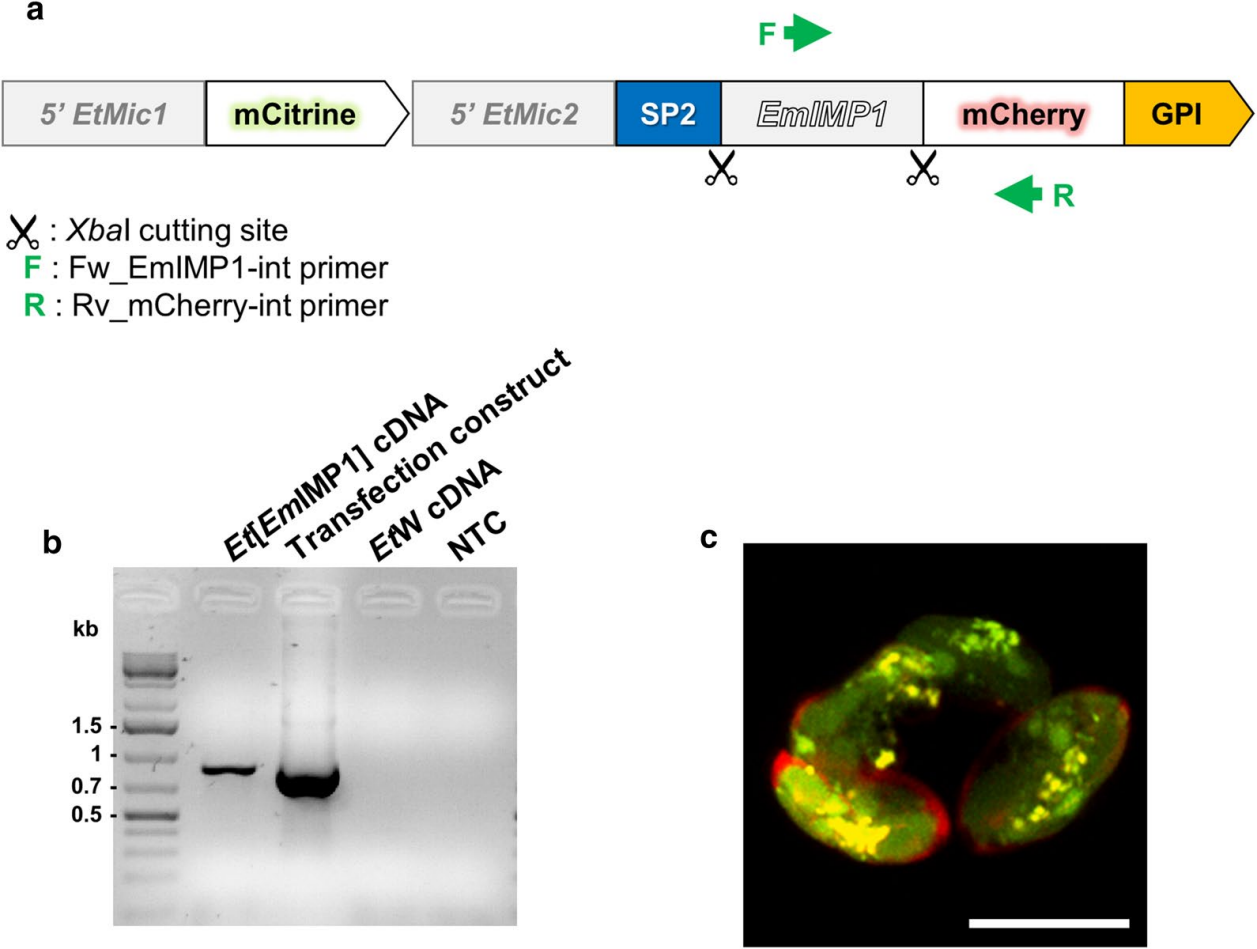
Preparation of transgenic *Eimeria tenella* Wis parasites expressing *Em*IMP1. **a** Simplified representation of the plasmid used for *E. tenella* transfection coding for the *Em*IMP1 protein. Scissors represent the location of the *Xba*I restriction site used for transgene insertion. F and R represent the primers used to confirm transgene transcription by reverse transcription (RT)-PCR. **b** Detection of *EmIMP1-mCherry* transcripts in cDNA isolated from stable transgenic populations by RT-PCR. A single band of ~ 0.9 kb was obtained from *E. tenella* populations expressing *EmIMP1* (*Et*[*Em*IMP1]), but not from the wild-type vector (*Et*W). The construct used for parasite transfection was included as a positive control. A non-template control (NTC) was also included. **c** Detection of *Em*IMP1-mCherry expression by confocal microscopy. The mCitrine was expressed as a cytosolic protein and used to select transgenic parasites by flow cytometry, whereas the *Em*IMP1-mCherry fusion protein was secreted into the sporocyst cavity and anchored onto the sporozoite surface [17]. *Scale-bars*: 10 µm

Transgenic parasites expressing the *Em*IMP1 protein were stabilized by four successive *in vivo* passages under pyrimethamine selection followed by FACS enrichment of mCitrine expressing parasites. This resulted in 37% of the population expressing both reporters (mCitrine and mCherry, fused to *Em*IMP1; Fig. 1a) with efficiencies of FACS recovery close to 96%. *Em*IMP1 mRNA transcription was confirmed by RT-PCR in stabilized populations in the absence of gDNA contamination (Fig. 1b). *Em*IMP1 protein expression was indicated by detection of the *Em*IMP1-mCherry fusion protein by fluorescence microscopy, which was secreted into the sporocyst cavity and anchored onto the sporozoite surface as expected (Fig. 1c) [11, 17].

### Vaccine safety

Individual body weights were recorded before vaccination (2 days of age) and before challenge (29 days of age). Statistical analysis of average body weights at day 2 demonstrated that chicks were evenly distributed between groups (ANOVA: *F*_(5, 144)_ = 0.5109, *P* = 0.7677). Analysis of BWG from 2 to 29 days of age showed that vaccination with live transgenic *E. tenella* parasites was not detrimental in terms of growth, as all groups performed equally (Kruskal-Wallis test: *χ*^2^ = 10.08, *df* = 5, *P* = 0.0731). Viability of vaccine lines was confirmed by faecal flotation. Faeces collected from the bottom of all cages 8 days after each immunisation, displayed varying numbers of non-sporulated oocysts, confirming that vaccine lines were cycling (data not shown). In all analyses, non-vaccinated birds remained uninfected.

### Effect of vaccination on parasite replication and local immune responses

**Fig. 2:**
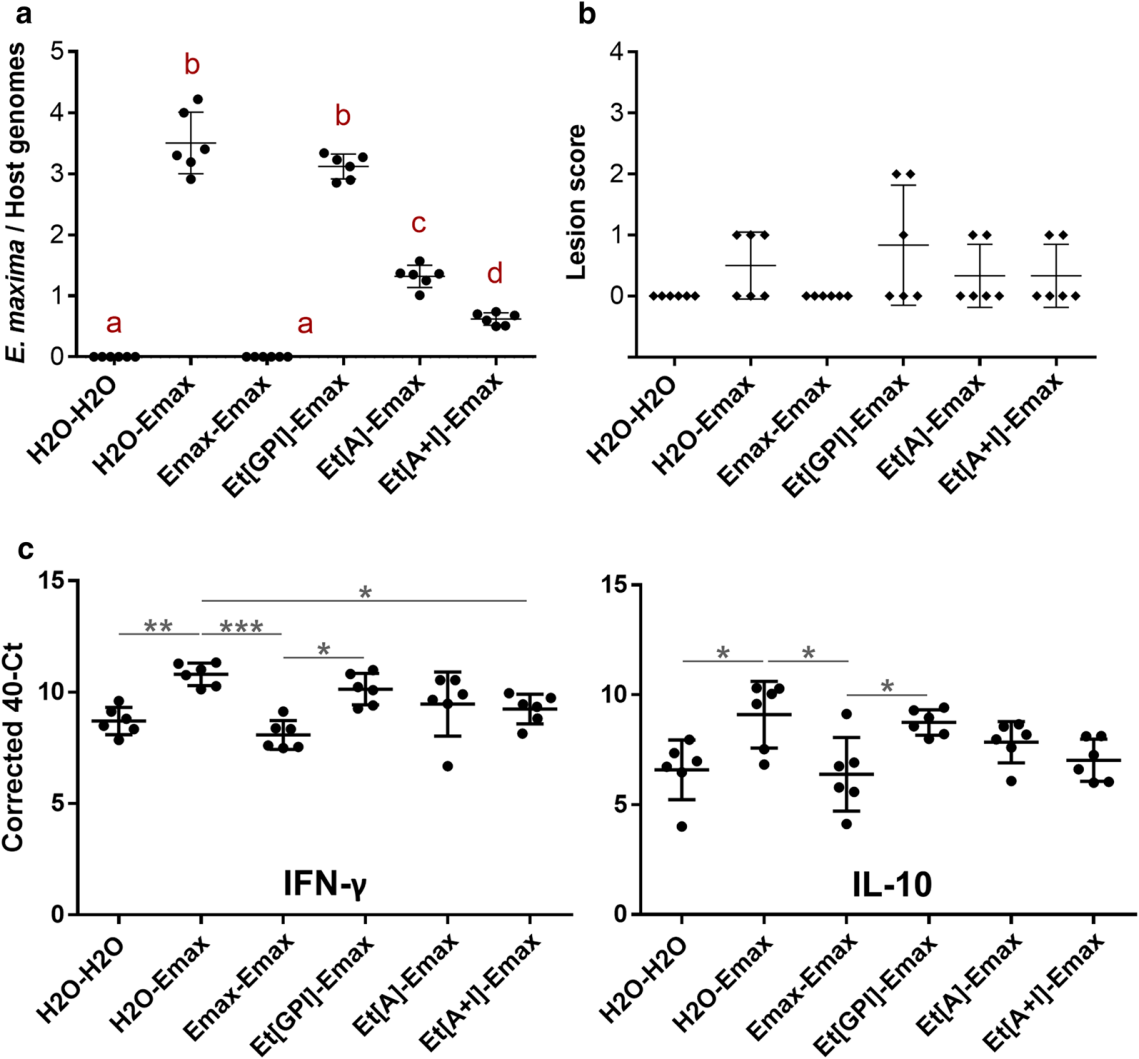
Vaccine efficacy against low *E. maxima* W challenge (300 oocysts/bird). **a***Eimeria maxima* W burdens quantified by q-PCR and presented as a parasite genomes per host genome ratio. Dots represent individual animals and bars indicate average values and standard deviations. Groups marked with different letters were significantly different (ANOVA, *P* < 0.0001). **b** Lesion scores observed in chickens used to quantify parasite replication. Diamonds represent individual animals and bars indicate average values and standard deviations. No differences were observed (Kruskal-Wallis test, *P* = 0.3803). **c** IFN-γ and IL-10 local immune responses in the intestine from birds used to quantify parasite replication. Dots represent individual animals and bars indicate average values and standard deviations. Groups linked with lines were significantly different (ANOVA, *P < 0.05, **0.0001 < *P* < 0.05, ***P < 0.0001)

In order to assess the efficacy of vaccination after a deliberately low challenge, replication of *E. maxima* W parasites was quantified by q-PCR in DNA samples extracted from the mid-point of the intestine [26]. Non-vaccinated and non-challenged birds (H_2_O-H_2_O), together with birds vaccinated and challenged with *E. maxima* W (Emax-Emax), did not display any evidence of parasite replication. On the contrary, non-vaccinated and challenged birds (H_2_O-Emax) and birds vaccinated with the empty vector (*Et*[GPI]-Emax) displayed the highest replication scores. Chickens vaccinated with *Et*[*Em*AMA1] (*Et*[A]-Emax) and the combination of *Et*[*Em*AMA1] and *Et*[*Em*IMP1] parasites (*Et*[A + I]-Emax) displayed a significant reduction in parasite replication compared to the non-vaccinated and challenged group (H_2_O-Emax), and to the group vaccinated with the empty vector (*Et*[GPI]-Emax) (ANOVA: *F*_(5, 30)_ = 254.1, *P* < 0.0001) (Fig. 2a). This reduction was more pronounced in the *Et*[*Em*AMA1] + *Et*[*Em*IMP1] group, where parasite replication was also significantly lower than that of the group vaccinated with *Et*[*Em*AMA1] alone (ANOVA: *F*_(5, 30)_ = 254.1, *P* = 0.0002) (Fig. 2a). While differences observed in parasite replication did not have any impact on body weight gains from 29 to 35 days of age (before challenge and 6 days post-challenge) in any groups in these low dose challenged chickens (Kruskal-Wallis test: *χ*^2^ = 6.374, *df* = 5, *P* = 0.2715), birds from both vaccinated groups displayed lower lesion scores than those non-vaccinated or vaccinated with the empty vector, although no statistical differences were found (Kruskal-Wallis test: *χ*^2^ = 7.926, *df* = 5, *P* = 0.1603) (Fig. 2b). As expected, the low challenge dose used to quantify parasite replication was not adequate to induce differences in BWG or lesion scores.

Intestinal samples were also used to analyse local transcription levels of IFN-γ and IL-10 by q-PCR after challenge. Overall, higher differences were observed in IFN-γ levels between groups: the highest IFN-γ levels were observed in non-vaccinated birds (H_2_O-Emax) and in those vaccinated with the empty vector (*Et*[GPI]-Emax), illustrating a typical primary response against *E. maxima* W. By contrast, birds vaccinated with *E. maxima* W (Emax-Emax) did not mount an IFN-γ response after homologous challenge, indicating a secondary response against homologous challenge. Interestingly, birds vaccinated with the *Et*[*Em*AMA1] + *Et*[*Em*IMP1] combination (*Et*[A + I]-Emax) showed lower IFN-γ levels compared with the non-vaccinated and challenged group (H_2_O-Emax), suggesting a secondary-type response against *E. maxima W* (ANOVA: *F*_(5, 30)_ = 8.426, *P* = 0.0289). Birds vaccinated with *Et*[*Em*AMA1] alone (*Et*[A]-Emax) did not show clear differences with any control group (H_2_O-H_2_O, H_2_O-Emax, Emax-Emax or *Et*[GPI]-Emax), suggesting an intermediate primary-secondary response against the parasite (Fig. 2c). Regarding IL-10, mRNA levels were increased after *E. maxima* W challenge in non-vaccinated birds (H_2_O-Emax) and chickens receiving the empty vector (*Et*[GPI]-Emax) compared to birds vaccinated and challenged with *E. maxima* W (Emax-Emax) (ANOVA: *F*_(5, 30)_ = 5.08, *P* < 0.05). This was indicative of primary and secondary responses against *E. maxima* W, respectively. Interestingly, no statistical differences were found with any vaccinated group, which could also indicate an intermediate response in those animals (Fig. 2c). In addition, when we performed correlation tests, they showed a positive correlation for both IFN-γ and IL-10 with parasite replication scores (Two-tailed Pearson’s test: *r*_(42)_ = 0.6817, *P* < 0.0001 for IFN-γ; *r*_(42)_ = 0.6175, *P* < 0.0001 for IL-10).

### Effect of vaccination on production scores and pathology

A total of 90 birds (18 per group) were challenged with a high dose of *E. maxima* W (10,000 oocysts) to assess the efficacy of vaccination with transgenic parasites against development of local lesions (7 birds per group, determined 6 days after challenge) and against reduced body weight gain (11 birds per group, calculated 11 days after challenge). Eighteen additional birds were not challenged and served as negative controls.

**Fig. 3:**
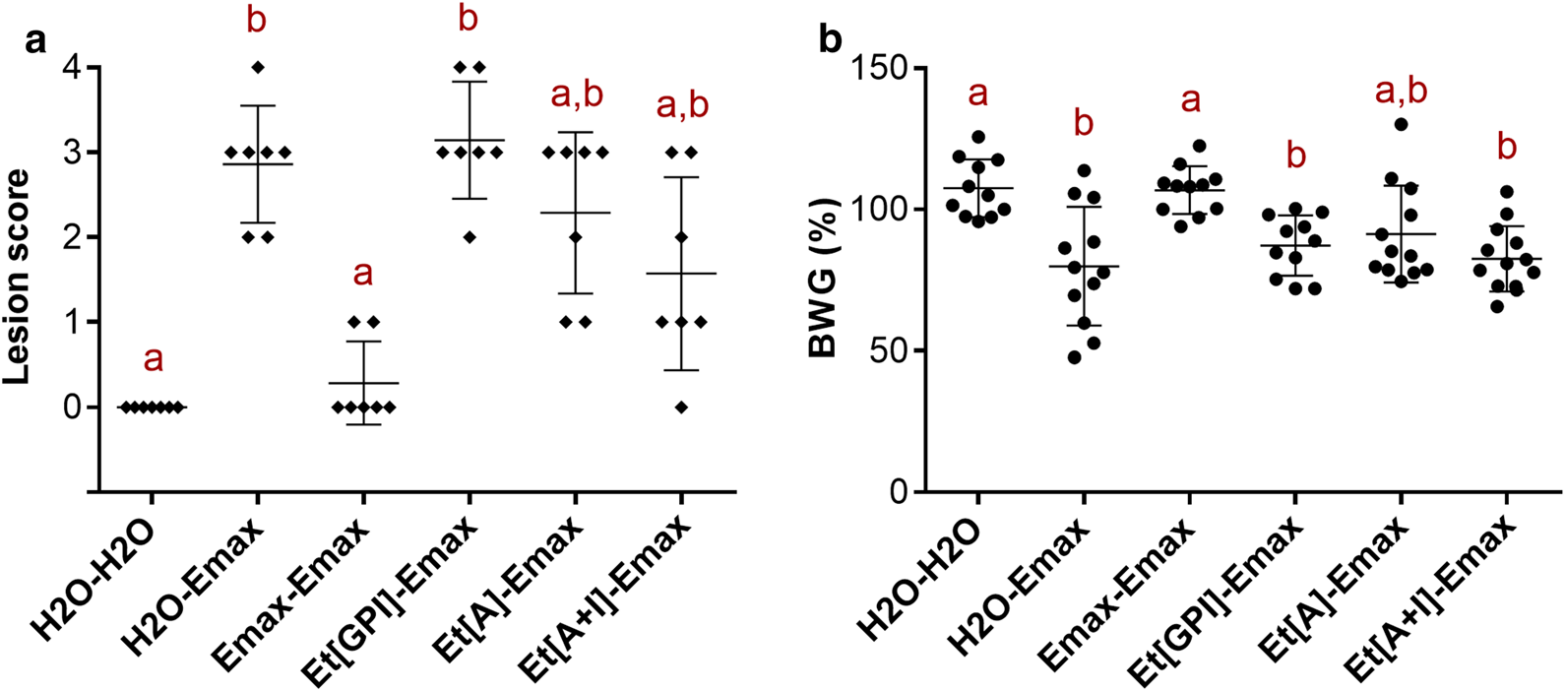
Vaccine efficacy against high *E. maxima* W challenge (10,000 oocysts/bird). **a** Intestinal lesion scores from vaccinated and control chickens. Lesion scores were determined 6 days after *E. maxima* W challenge (35 days of age). Diamonds represent individual animals and bars indicate average values and standard deviations. Groups marked with different letters were significantly different (Kruskal-Wallis test, *P* < 0.05). **b** Percentage body weight gains (BWG) from vaccinated and control chickens 12 days after challenge. BWG was calculated from day of challenge (29 days of age) to day of cull (41 days of age). Dots represent individual animals and bars indicate average values and standard deviations. Groups marked with different letters were significantly different (ANOVA, *P* < 0.05)

The distribution of lesion scores among groups is shown in Fig. 3a. Vaccination with *E. maxima* W parasites (Emax-Emax) yielded the best protection results, showing no statistical differences with the non-challenged birds (H_2_O-H_2_O) (Kruskal-Wallis test: *χ*^2^ = 30.57, *df* = 5, *P* > 0.05) as only two out of seven animals showed lesions, both of which were very mild. Conversely, non-vaccinated birds (H_2_O-Emax) and those immunised with the empty vector (*Et*[GPI]-Emax) displayed the highest lesion scores, showing clear differences with the non-challenged birds (H_2_O-H_2_O) (Kruskal-Wallis test: *χ*^2^ = 30.57, *df* = 5, *P* < 0.05). Vaccination with *Et*[*Em*AMA1] alone (*Et*[A]-Emax) or the *Et*[*Em*AMA1] + *Et*[*Em*IMP1] combination (*Et*[A + I]-Emax) reduced the average lesion scores but statistically there were no differences between these and either the non-protected (H_2_O-Emax and *Et*[GPI]-Emax groups) or the ‘fully’ protected (Emax-Emax) groups (Kruskal-Wallis test: *χ*^2^ = 30.57, *df* = 5, *P* > 0.05). Interestingly, average lesion scores were lower in the group vaccinated with *Et*[*Em*AMA1] + *Et*[*Em*IMP1] parasites, with the majority of birds showing lesion scores under 2; however, these differences were not significant (Kruskal-Wallis test: *χ*^2^ = 30.57, *df* = 5, *P* > 0.05).

Percentages of BWG are displayed in Fig. 3b. Similarly to the lesion scores, chickens vaccinated with *E. maxima* W oocysts (Emax-Emax), performed as well as non-challenged birds (H_2_O-H_2_O) (ANOVA: *F*_(5, 64)_ = 8.373, *P* > 0.05), whereas non-vaccinated and challenged birds (H_2_O-Emax) and birds vaccinated with the empty vector (*Et*[GPI]-Emax) showed significant reductions in BWG (ANOVA: *F*_(5, 64)_ = 8.373, *P* < 0.05). Neither *Et*[*Em*AMA1] nor *Et*[*Em*AMA1] + *Et*[*Em*IMP1]-vaccinated groups showed statistical differences to the ‘non-protected’ groups (H_2_O-Emax and *Et*[GPI]-Emax), suggesting that vaccination was insufficient to prevent body weight losses (ANOVA: *F*_(5, 64)_ = 8.373, *P* > 0.05). However, birds vaccinated with *Et*[*Em*AMA1] did not display any significant difference from the ‘fully protected’ animals (Emax-Emax) and the non-challenged birds (H_2_O-H_2_O) either, indicating that this formulation was able to induce partial levels of protection against reduced body weight gain (ANOVA: *F*_(5, 64)_ = 8.373, *P* > 0.05). Since variability in the H2O-Emax group was very high, removal of the outlier individuals for supplementary statistical analysis resulted in three clear clusters of animals: ‘fully protected’ (H_2_O-H_2_O and Emax-Emax); ‘non-protected’ (H_2_O-Emax); and ‘partially protected’ (*Et*[GPI]-Emax, *Et*[A]-Emax and *Et*[A + I]-Emax) (ANOVA: *F*_(5, 61)_ = 13.72, *P* < 0.05) (data not shown).

## Discussion

Live anticoccidial vaccines are highly effective for control of poultry coccidiosis caused by *Eimeria* spp., but their price and limited availability preclude broad usage across much of the broiler sector where anticoccidial drugs are still dominant. Anticoccidial vaccine candidates are available as the basis of future subunit vaccines, but strategies for effective and scalable delivery are yet to be established. In response, studies have been focused on the development and validation of genetically modified *E. tenella* parasites expressing antigens from other *Eimeria* species with the aim of (i) establishing an automated single-shot delivery system suitable for intensive farming systems, (ii) inducing significant levels of immune protection against different *Eimeria* species, and ultimately (iii) simplifying current vaccine formulations from 7–8 parasite lines to a small number of transgenic *Eimeria* populations expressing antigens from different *Eimeria* species. We and others have previously demonstrated that *E. tenella* can express exogenous reporter genes [27, 28], antigens of other poultry pathogens [20, 29], and also vaccine candidates from other *Eimeria* species such as *E. maxima* [11–13]. These later publications have highlighted the efficacy of *E. tenella* parasites expressing *Em*AMA1 and *Em*IMP1 as vaccines that can protect against *E. maxima* challenge of inbred chickens. For this study we aimed to reassess the efficacy of these vaccines in a more commercially relevant scenario of poultry coccidiosis, mimicking an intensive farming system where broiler breeds are reared at high densities and risk exposure to high levels of *Eimeria* oocysts. Knowing in advance that these vaccines were not able to induce sterile protective immunity, we focused our interest on determining if vaccination was sufficient to prevent reduced body weight gain and/or severe gut pathology at levels that could be acceptable from a commercial perspective.

Prior to *E. maxima* challenge growth performance was comparable between vaccinated and non-vaccinated chickens, supporting our previous findings with regards to vaccine safety [11]. Notably, vaccination with *Et*[*Em*AMA1] or with the combination of *Et*[*Em*AMA1] and *Et*[*Em*IMP1] conferred significant protection against *E. maxima* replication, with chickens displaying a significantly reduced *E. maxima*/host genomes ratio, especially in those receiving the bivalent vaccine. These results confirm observations using inbred chicken lines where vaccination with *Et*[*Em*AMA1], *Et*[*Em*IMP1], or *Et*[*Em*AMA1] plus *Et*[*Em*IMP1] significantly reduced total oocyst outputs after challenge with low *E. maxima* doses [11–13, 18].

Vaccination with transgenic *E. tenella* that expressed *E. maxima* antigens modified the host immune response against subsequent *E. maxima* challenge. It is well established that resistance to primary *Eimeria* infection is mediated by IFN-γ [30–32]. In the case of *E. maxima*, previous studies have described the occurrence of different local immune responses after challenge, with IFN-γ mRNA levels peaking after the first infection and being almost unaffected by subsequent infections [33]. We observed the same response in Emax-Emax chickens after secondary infection (low IFN-γ mRNA levels, similar to those observed in H_2_O-H_2_O chickens) compared to primary infection in H_2_O-Emax birds (high IFN-γ mRNA levels). However, birds vaccinated with transgenic parasites (*Et*[A]-Emax and *Et*[A + I]-Emax) showed intermediate IFN-γ mRNA responses, indicating the development of a certain degree of immune memory against *E. maxima*. This finding is supported by a previous study, where vaccination with *Et*[*Em*AMA1] + *Et*[*Em*IMP1] was enough to induce specific IFN-γ responses after stimulation of PBMCs with *E. maxima* extracts [13]. We also measured IL-10 levels in the intestine since this cytokine has been correlated with susceptibility to *E. maxima* infection, possibly through inhibition of IFN-γ synthesis [34, 35]. Local IL-10 mRNA levels showed a pattern similar to that described for IFN-γ, with low levels of expression in the Emax-Emax group, high levels in the H_2_O-Emax group and intermediate levels in the *Et*[A]-Emax and *Et*[A + I]-Emax groups. This is in agreement with our previous study, where IL-10 serum levels were significantly lower in birds vaccinated with transgenic *Et*[*Em*AMA1] parasites after *E. maxima* challenge compared to non-vaccinated and challenged birds [11]. This reduction in intestinal IL-10 levels could favour the development of IFN-γ-mediated responses, with effective immune killing of replicating parasites and a consequent reduction of oocyst shedding as previously suggested [34, 35].

It has previously been shown that (i) quantification of oocyst shedding following a low dose challenge is not an appropriate indicator of protection against clinical coccidiosis (normally induced by significantly higher numbers of parasites), and (ii) that infection with higher doses would increase oocyst shedding with little or no correlation to growth performance [36]. For this reason, we also challenged a group of chickens with higher doses of sporulated *E. maxima* oocysts (10,000 per bird) and culled them at two different time-points to assess the effect of vaccination on gut lesions (6 days after challenge) and BWG (12 days after challenge). Severity of gut lesions was partially reduced by vaccination with *Et*[*Em*AMA1] and this effect was slightly better when *Et*[*Em*AMA1] and *Et*[*Em*IMP1] parasites were combined. Chickens vaccinated with any of the formulations displayed intermediate lesion scores that did not differ from those observed in the ‘fully protected’ or ‘unprotected’ control groups. This phenomenon might be an effect of the enhanced IFN-γ responses triggered by vaccination as suggested for *E. tenella* infections [37]. While a reduction in gut pathology following challenge can be taken as a proof of protection by anticoccidial vaccines, it has been demonstrated that the use of lesion scores alone may underestimate efficacy since commercially vaccinated chickens with lesions are able to perform as well as birds with no lesions in terms of BWG [38, 39].

Performance parameters such as BWG remain a key accepted criterion to evidence effective development of protective immune responses in vaccinated chickens after high-level *Eimeria* challenge [36]. In our trial, only vaccination with *Et*[*Em*AMA1] parasites was able to induce partial levels of protection, with birds showing intermediate performance compared to the ‘fully protected’ and ‘unprotected’ control groups. We observed the same effect in a previous pilot trial where Cobb500 birds were vaccinated once with 100 *Et*[*Em*AMA1] oocysts (data not shown). Similarly, vaccination with AMA1 from varying *Eimeria* species using diverse vaccine platforms has been shown to be able to confer partial levels of protection against reduced weight gain after high-level homologous parasite challenge [40–42]. Intriguingly and despite evidence that vaccination using IMP1 can induce protection in terms of BWG [43–45], we did not observe any notable protection in growth of chickens vaccinated with *Et*[*Em*AMA1] + *Et*[*Em*IMP1]. Differences in growth performance between the *Et*[*Em*AMA1] and *Et*[*Em*AMA1] + *Et*[*Em*IMP1] vaccinated groups may be a consequence of the differential antigen load of each formulation, since the *Et*[*Em*AMA1] + *Et*[*Em*IMP1] group was immunised with half the number of *Em*AMA1-expressing parasites compared to the *Et*[EmAMA1] group. This suggests that antigen load should be always considered as it may influence the presence or absence of a protective response, at least in terms of BWG. However, since the parasite populations used for immunisation were not clonal it is extremely difficult to determine the exact quantity of transprotein that was effectively delivered in each vaccine formulation, even employing indirect methods such as the q-PCR described earlier [15]. It is also worth highlighting that the variation observed in BWG in the non-vaccinated and challenged control group could have interfered with data interpretation, since performance of a quarter of those chickens was comparable to birds from the non-challenged (H_2_O-H_2_O) and the vaccinated (Emax-Emax) control groups. This variation likely reflects individual differences in susceptibility to coccidiosis, mainly attributed to breeding programs in hybrid commercial chicken lines [46, 47]. For this reason, broilers should not be used to test vaccine efficacy of new formulations in the first instance [36].

## Conclusions

Here we confirm that vaccination of commercial broiler chickens with *E. tenella* parasites expressing *Em*AMA1, or the combination *Em*AMA1 + *Em*IMP1, is able to significantly reduce *E. maxima* replication following subsequent challenge. The level of protection was higher when both antigens were combined. We also show that vaccination using these transgenic parasite lines partly modifies host immune responses against heterologous *E. maxima* challenge, at least in terms of local IFN-γ and IL-10 responses, which could lead to earlier immune recognition and reduction of parasite replication. Vaccination with both formulations also reduced the severity of pathology after high level challenge, with *Et*[*Em*AMA1] + *Et*[*Em*IMP1] showing the lowest average lesion scores correlated with a reduction in parasite replication. Nonetheless, only chickens vaccinated with *Et*[*Em*AMA1] parasites were partially protected against reduced body weight gain, although the high levels of variation observed in the non-vaccinated and challenged control groups prevented robust comparison. Overall, the results of this work offer good prospects for future development of multivalent anticoccidial vaccines for commercial systems using appropriate vaccine candidates. Thus, our efforts should now be focused on the discovery of optimal targets for vaccination and their validation and assessment to exploit the opportunities of this toolbox.

## Data Availability

All data generated during this study are included in this published article. Additional information is available from the corresponding author upon reasonable request.
